# Assessing changes in HIV-related legal and policy environments: Lessons learned from a multi-country evaluation

**DOI:** 10.1371/journal.pone.0192765

**Published:** 2018-02-23

**Authors:** Laura Ferguson, Alexandra Nicholson, Ian Henry, Amitrajit Saha, Tilly Sellers, Sofia Gruskin

**Affiliations:** 1 Program on Global Health and Human Rights, Institute for Global Health, University of Southern California, Los Angeles, California, United States of America; 2 HIV, Health and Development Team, United Nations Development Program, Istanbul Regional Hub, Istanbul, Turkey; 3 Health and Development Team, United Nations Development Program, Regional Centre for Africa, Addis Ababa, Ethiopia; Yale University Yale School of Public Health, UNITED STATES

## Abstract

**Introduction:**

There is growing recognition in the health community that the legal environment—including laws, policies, and related procedures—impacts vulnerability to HIV and access to HIV-related services both positively and negatively. Assessing changes in the legal environment and how these affect HIV-related outcomes, however, is challenging, and understanding of appropriate methodologies nascent.

**Methods:**

We conducted an evaluation of a UNDP project designed to strengthen legal environments to support the human rights of key populations, in particular LGBT populations, women and girls, affected by HIV in sub-Saharan Africa. We analyzed data on activities designed to improve legal environments through a systematic document review and 53 qualitative interviews.

**Results:**

The project made substantial strides towards legal change in many places, and examples provide broader lessons for work in this area. Two core pillars appear fundamental: a government-led participatory assessment of the legal environment, and building the capacity of those impacted by and engaged in this work. Systematic attention to human rights is vital: it can help open new spaces for dialogue among diverse stakeholders, foster new collaborations, and ensure local ownership, nuanced understanding of the political landscape, attention to marginalized populations, and accountability for (in)action. Entry points for effecting legal change go beyond “HIV laws” to also include other laws, national policies and strategies.

**Conclusion:**

Conducting legal environment assessments, multi-stakeholder dialogues, action planning and related activities, alongside capacity building, can contribute to changes in knowledge and attitudes directly relevant to reforming laws that are found to be harmful. Shorter-term goals along the causal pathway to legal change (e.g. changes in policy) can constitute interim markers of success, and recognition of these can maintain momentum. Increasing understanding of progress towards changes in the legal environment that can positively affect HIV-related outcomes is important in working to improve the health and lives of people living with HIV.

## Introduction

### Background

There is growing recognition amongst those who work on HIV that the legal environment—including laws, policies, and related procedures—impacts vulnerability to HIV and access to HIV-related services in myriad ways both positive and negative [1]. For example, non-discrimination provisions in laws may protect people living with HIV (PLHIV) or other vulnerable or marginalized populations from discrimination and offer legal means of redress should discrimination occur, allowing people to more openly access HIV-related services [1]. Conversely, laws can increase vulnerability to HIV and decrease access to services such as laws prohibiting access to needle exchange programs, which may promote needle-sharing among injecting drug users [2,3], or laws criminalizing same-sex sex, which may inhibit men who have sex with men (MSM) from accessing health services [4]. Human rights frameworks enable analysis of legal environments, with such analyses underscoring states’ legal obligations to respect, protect, and fulfil human rights in their HIV responses [5]. Widespread concurrence that obstructive laws can exacerbate the HIV epidemic and beneficial laws can ameliorate some of its effects and reduce disease incidence has driven political interest from countries around the world in the links between human rights, legal environments, and HIV responses [6].

Work recognizing the vital connections between legal environments and HIV responses is occurring at multiple levels. The Global Commission on HIV and the Law (the Global Commission) undertook extensive work to examine links between legal environments and HIV responses, which resulted in a comprehensive set of recommendations now driving action [7]. The current Global Fund to Fight AIDS, Tuberculosis and Malaria strategy includes explicit recognition that human rights-based approaches (HRBAs), directly linked to legal environments, increase the effectiveness, efficiency and sustainability of its programming [8–10]. Civil society also recognizes synergies between human rights, legal environments, and HIV, e.g. Open Society Foundations promotes human rights and access to justice to safeguard the health of all people, including vulnerable populations such as PLHIV [11].

Assessing changes in the legal environment and how these affect HIV-related outcomes, however, is challenging, and understanding of appropriate methodologies to do so remains nascent. The causal pathway is long and complex, creating challenges including with regard to attribution. Large-scale alterations in law rarely occur quickly, and frequently not within project periods or funding cycles. A mix of carefully pieced together quantitative and qualitative data is undoubtedly required.

This article explores what it takes to support HIV-related legal change and some of the challenges inherent in doing so. It draws on lessons from evaluating a Sida-funded project implemented by the United Nations Development Programme (UNDP). We analyzed data on activities designed to contribute towards HIV-related legal change, identifying potential interim indicators along the pathway to legal change that can be used across different legal, political and health systems and HIV epidemics. Examples from the project illustrate broader lessons.

### The project

UNDP implemented a three-year project (2013–5), based on the Global Commission’s recommendations for improving HIV-related legal environments, in 11 countries in sub-Saharan Africa (The Democratic Republic of the Congo, Ghana, Kenya, Lesotho, Malawi, Mozambique, the Seychelles, South Africa, Swaziland, Tanzania and Zambia). The project, “Strengthening Regional and National Legislative Environments to Support the Human Rights of LGBT People and Women and Girls affected by HIV and AIDS in Sub-Saharan Africa,” was tailored to local contexts and set up to be responsive to the different needs of project countries. The project was designed and implemented with a focus on rights principles, specifically participation and inclusion, equality and non-discrimination, and accountability, key elements of a HRBA to health [12].

The project sought to effect change in challenging legal environments so as to increase access to justice and improve HIV-related outcomes. It operated at multiple levels—project, sub-regional, national and sub-national–across diverse political, legal, epidemiological, and cultural settings. Stemming from recognition that existing approaches to addressing law and human rights in national HIV responses in sub-Saharan Africa were inadequate and insufficiently supportive of the rights and health of key populations [12], the project developed an approach to support national actors to conduct Legal Environment Assessments (LEAs) to take stock of laws, policies and practices that affect PLHIV, key and vulnerable populations, as well as National Dialogues (NDs) to bring together key stakeholders to share LEA findings, prioritize areas for advocacy and action, and create action plans for legal reform [12]. This approach was intended to facilitate national and regional level consensus on next steps to address legal change in order to positively impact HIV, in particular among key populations [12].

## Methods

From September 2014 to December 2015 we conducted an evaluation focused on assessing the overall effects of the project described above. We sought to identify enabling factors and challenges, and to reach conclusions concerning the project’s contribution to change so as to provide actionable recommendations for improvements [13]. We analyzed data on activities designed to contribute towards legal change with a view to identifying potential areas where interim indicators along the pathway to legal change could be used.

Given the project’s grounding in rights, human rights served as the evaluation’s cornerstone to highlight the rights dimensions in how the project was implemented; particular attention was given to the principles of inclusion, participation, equality and non-discrimination, and accountability that the project had stated were central to its work. With these principles in mind, the evaluation focused on project implementation processes in a conscious attempt to better understand the extent to which these principles were incorporated into how activities were designed, carried out, monitored and evaluated. This included, for example, seeking to understand to the extent possible who has led the project processes at each level, and which partners were engaged in different phases of the project.

Key global, regional and national project documents were reviewed using a Data Extraction Tool, which was modified for different document categories and allowed for cohesive thematic analysis across documents. Qualitative data were collected through 53 semi-structured interviews with project stakeholders, including representatives of government, civil society organizations, key populations, United Nations agencies and others, across a range of countries. Standardized interview guides were used to ensure consistency, connection to the document review, and quality, breadth and depth of data. Interviews focused on stakeholders’ involvement in and perceptions of work carried out; ongoing challenges in relation to the HIV-related legal environment for key populations, particularly for LGBT populations, women and girls; and priorities for future work. Outputs from the document review and qualitative interviews were thematically analyzed together with a priori themes drawn from the project’s conceptual framework and the key human rights principles noted, which were supplemented by emergent themes from the data.

In-depth case studies involving desk reviews and primary data collection were carried out in Malawi and the Democratic Republic of the Congo (DRC) to further explore implementation processes and how to maximize project contributions towards desired outcomes and sustainable change. These countries were selected to reflect diversity in legal systems, political systems, epidemiology, national responses to HIV, as well as the breadth and depth of lessons of relevance to the project.

This work was a multi-country program evaluation focused entirely on the legal and policy environment and not human subjects research. All interview participants belonged to organizations formally involved in the program under evaluation; it was therefore determined that informed consent was not required. Attention to ethical considerations remained paramount throughout qualitative data collection: participants were fully informed about the evaluation, and all participation was voluntary and data were anonymized before write-up.

## Results

This section presents key evaluation findings, including smaller-scale project achievements within countries that are critical to understanding interim steps towards legal change.

By mid-term, the project resulted in four LEAs, four NDs, and capacity building activities or implementation plans in all project countries. Additional LEAs and NDs were also being planned.

Although the project had not effected explicit legal change within its first 18 months, it had made substantial strides towards this in most places. In many countries, these activities may have been as important as the legal change originally driving the project. Some of the common steps along the pathways to legal change were part of project design, and some were interim outputs/outcomes.

Two core pillars of the project appear fundamental: a government-led participative assessment of the legal environment, and building the capacity of those impacted by and engaged in this work. Further, using a HRBA is of key importance.

Across all countries, several lessons merit attention with respect to the project and to legal change more broadly. These are explored below.

### Assessment of the legal environment

The LEAs brought together a wide range of stakeholders and provided an overview of national legal environments. LEAs identify and examine national and sub-national HIV-related legal and human rights issues, often culminating in a ND where preliminary findings are discussed and validated. Stakeholder consultations (ranging from policymakers to representatives of key populations) and desk reviews of national laws, regulations and policies using human rights standards as a framework for review are combined to obtain the necessary information [14]. The process of conducting LEAs is critical: driven by national governments with appropriate technical support from external consultants with extensive substantive and procedural knowledge, their participative nature can engender broad local ownership and bring together stakeholders who may have never previously collaborated. For example, in Ghana the ND was used as an opportunity to bring together government representatives, parliamentarians, law enforcement, the judiciary, and more, to engage with key populations and civil society on rights-based issues related to the country’s HIV response [5]. In the DRC, the ND was the first time that representatives of the MSM community had ever spoken out about issues relating to their health and lives with members of the Parliament, representatives of the Ministry of Justice, and other government stakeholders [5]. A challenge in this work was ensuring that members of key populations felt able to articulate their experiences and concerns in these fora, and substantial capacity building was carried out in some countries to facilitate this. In some countries, there was limited participation by bisexual and transgender populations, perhaps reflecting the fact that these groups are less organized and visible than, for example, MSM and sex workers.

The assessments resulting from the ND can provide a critical summary of HIV-related legal environments that is credible to local stakeholders as a platform for prioritizing actions amongst diverse stakeholders to promote an enabling legal environment. With sufficient local traction, high-level political leadership can be engaged. For example, in Malawi, the LEA concluded that early marriage should be prohibited and harmful cultural and religious practices that increase HIV risk should be reviewed with a view to prohibition. The national Parliament has since passed a Bill, which has received Presidential assent, prohibiting child marriage and raising the universal age of marriage to 18 years for both girls and boys [15]. While a Constitutional Referendum may be required to formalize this change, and implementation at local level will require attention to a host of factors, it remains a significant advance.

In many countries, LEAs generated recognition of the need to create legal protection for key populations against discrimination on the basis of real or perceived HIV status as well as the multiple layering of stigma and discrimination that is experienced across these groups. For example, the LEA from the Seychelles recommended that the State may “wish to consider an amendment to the Constitution to include HIV as a prohibited ground of non-discrimination,” and strongly emphasized the need to bolster the capacity of health workers to attend to vulnerable populations [16]. The Lesotho LEA concluded that the national legal environment has “not yet addressed specific challenges facing key populations and people most at high risk of HIV transmission” and that stigma and discrimination exacerbated the negative impact of HIV among key populations, including their access to services [16]. Here too the LEA is generating follow-up activity amongst diverse partners.

Another common area of increased understanding was the need to repeal discriminatory laws such as inheritance laws, laws affecting access to HIV-related services, laws criminalizing consensual sex between adults, overly broad laws criminalizing HIV exposure or transmission, and laws criminalizing consensual sex work. For example, the Lesotho LEA recommended that Lesotho not criminalize HIV transmission or exposure, as malicious or intentional transmission or exposure could be addressed through existing legislation [17].

Across most countries, the LEA process generated explicit focus on increasing equality in access to services and reducing stigma and discrimination. For example, there were reports from the DRC that some MSM seek HIV services in Burundi due to the discrimination that they face in health services within the DRC; the need to address this was highlighted. The Lesotho LEA noted that stigmatizing attitudes in healthcare settings should be reduced and healthcare providers given the skills and tools to ensure the rights of all clients, including sex workers, to informed consent, confidentiality, treatment, and non-discrimination [17].

### Capacity building

Capacity building activities are critical, occurring at different levels and taking many forms. Activities might focus on a specific type of stakeholder such as judges or law enforcement agents, or bring together diverse stakeholders to create shared understanding of an issue. They can: take place locally, nationally or at the regional level; include the creation of guidelines, training materials or briefs; or include support for lobbying for legal change, with civil society and others highlighting to law-makers why this is required. The key issue is that capacity to understand and address the impacts of the legal environment on HIV-related prevention, care and treatment remains low in most places. Any capacity building should pay explicit attention to the need for understanding the LEA process, its findings and their impact on the country’s HIV epidemic.

In the DRC, there was recognition that the justice system was inadequately prepared to protect people who experienced HIV-related human rights violations. There was a need to help people learn how to report violations accurately and put together a valid case, as well as to ensure a functioning court system with sufficient training to hear cases fairly. The Ministry of Justice was central in trying to address this including through the ND process, which led to the organization of parliamentary fora on HIV, establishment of a multi-stakeholder observatory on HIV-related human rights, and plans for training judges, lawyers and activists in six cities. These were widely recognized as useful first steps, replicable elsewhere, towards building capacity to address system weaknesses with ongoing efforts required to fully address the issues and engender confidence in the formal legal system.

### Human rights in project implementation processes

How a project is implemented also influences its outcomes so due attention to relevant processes of implementation is required in any assessment. Systematic attention to human rights principles from the outset was another key to this project’s success, including the ability to foster local ownership, ensure attention to marginalized populations, and promote accountability. Table 1 outlines broad lessons learned from UNDP’s application of a HRBA with attention to each rights principle.

**Table 1:** The value of using human rights in project implementation.

Principles of a Human Rights-Based Approach (HRBA)	How Human Rights Principles were used in Project Implementation
Participation/ Inclusion	• Ensuring the perspectives of a wide range of stakeholders, including key populations, form the substance of the LEA. • Designing NDs to be inclusive and participative to create safe, open spaces where key populations, including women, girls and LGBT people, could engage in frank and constructive dialogue with those who write, shape, and enforce laws that impact on their lives. • Inclusion of a range of identified stakeholders, such as networks of PLHIV, those most at risk of HIV, and key populations, to foster inclusivity and better representation of the concerns of these populations in all aspects of project work.
Equality/ Non-Discrimination	• Evaluations of national legal frameworks and their implementation against international human rights standards, with a focus on discrimination and inequality in law and policy, and positive attention to the right to be free from discrimination, and the right to equality and equal protection from the law. • Emphasis on the inclusion of a wide range of key populations in discussions of national law, policy and practice to ensure lived experiences of discrimination are heard and understood also by those responsible for law implementation and enforcement.
Accountability	• Grounding the project in people’s empowerment including attention to tools to support their ability to hold institutions accountable, including project investment in strengthening access to justice alongside strengthening of leadership and capacity of regional and national actors. • Establishment of rights-oriented guidelines for LEA implementation as well as ND processes to guide actions to strengthen the legal and regulatory framework, as well as to empower rights-holders to claim their rights, and build the capacity of duty-bearers to implement rights.

These examples of how human rights principles were central to the processes of project implementation demonstrate not only good practice, but pragmatic approaches to using human rights to improve project outcomes.

## Lessons learned

Work on legal environments is challenging even in the best of circumstances. Several lessons learned through the use of human rights norms and standards in the implementation of this project are noted below for their potential relevance to other similarly motivated efforts.

### Opening space for dialogue

With a government-led, methodologically rigorous, comprehensive LEA and ND that bring together government, civil society and key populations, space can be created where previously taboo issues can be discussed in respectful, safe and constructive ways by actors not often in dialogue. These discussions can usefully be framed around international human rights commitments, which serves the dual purpose of providing legal grounding and increasing understanding of the country’s human rights commitments as they relate to HIV and more generally. Zambia, for example, reported that the ND process raised awareness among key stakeholders on the linkages between HIV, law and human rights and how the law can be relevant to the HIV response, including creating an enabling environment for PLHIV if compliant with human rights standards [18].

The participatory nature of these processes allowed for unprecedented discussion and commitment to examine and address relevant legal barriers, especially with regard to LGBT issues, and enabled the conversation to be tailored to local priorities. In the Seychelles, for example, the LEA was informed in part by focus group discussions purposely composed of a mix of populations vulnerable to and at high risk of exposure to HIV, including people who use drugs, prisoners, and young people [19]. These discussions resulted in the identification of specific areas of law negatively impacting the HIV response such as coercive public health measures. The need to enact general anti-discrimination legislation and amend health legislation that contained punitive measures for dealing with infectious disease was highlighted [19].

### New collaborations

Creating new spaces for dialogue amongst different actors opens the potential for new collaborations extending beyond envisioned project goals. In Ghana, for example, the ND was seen as “an opportunity to bring together representatives from government, the judiciary, law enforcement, parliamentarians, and the GAC [Ghana AIDS Commission] to engage meaningfully with civil society and affected populations on rights-based issues related to Ghana’s HIV/AIDS response [20].” The project ensured this served as the foundation for ongoing collaborations.

A multi-stakeholder technical working group in the DRC has been central to improving law and human rights issues relevant to HIV. The collaborative nature of the group allows for joint reflection on issues, the ability to offer multi-sectoral and multi- disciplinary training and support to government and civil society institutions, the ability to influence laws, policies, strategies and resource mobilization at the national level, and helps the Ministry of Justice to act as needed. Several respondents in the DRC noted that these sorts of interactions and collaborations between the Ministry of Justice, civil society and UN organizations are key to efforts to improve the HIV-related legal environment: “if you take away one of these three it will all collapse like a house of cards [21]”.

Building on these new spaces for dialogue and collaborations can result in shifts in knowledge and attitudes fundamental to effecting change in the law, especially when dealing with “sensitive” topics or topics where the links between law and HIV may not be well known locally.

### Availability of data on key populations

A recurring priority for this work is the need for more and better data on HIV, including on stigma, discrimination and the impacts of the legal environment on key populations. Data needs vary by country but extend across all key populations affected by HIV. Prior to the LEAs, issues relating to key populations had been little discussed in project countries, and bringing them to light highlighted the paucity of evidence to inform appropriate policies, including those that help to ensure access to quality information and services and do not discriminate. Both the DRC and Zambia underscored the need for better data to help direct HIV-related resources to key populations who need them. While there are currently a number of global efforts underway [22], collaborative efforts between key populations and governments at national-level are needed. One such effort in Malawi has produced useful data on MSM and HIV [23]. Integrated Biological and Behavioural Surveillance Surveys in some countries are beginning to shed some light on HIV prevalence and behaviours among sex workers and MSM, but there is still a glaring lack of data on bisexual and transgender populations in sub-Saharan Africa, as well as age-disaggregated data, especially for adolescent girls. Additional efforts are needed in this regard to help information national and subnational policies, plans, programmes and services.

### Targeting policies and strategies

In most countries, policy reform can present opportunities for collaborative work to effect real change in people’s lives in a relatively short timeframe, and serve as an evidentiary basis to support subsequent legal change.

In Malawi, the National HIV and AIDS Policy and Strategic Plan was revised as a result of the LEA to address religious and cultural values and norms that exacerbate gender inequality, discrimination and stigma towards key and vulnerable populations, and gaps in the legal framework to realize human rights in the context of HIV. In the DRC, ‘an enabling legal environment’ was included as a central axis of the national strategic plan for HIV as a result of advocacy efforts, building on awareness-raising activities that occurred through the ND and related processes. In addition, lubricant was added to the national Essential Medicines List as a direct result of advocacy by MSM at the ND. All of these actions are important in their own right, while also of key importance in providing data and precedent to support legal change efforts.

### Targeting legal reform

Even as many countries have an ‘HIV law’ containing problematic provisions requiring amendment this may not be the law with the most direct negative impact on people’s daily lives. A host of other laws can be identified through an LEA that may be of particular relevance for addressing HIV effectively. For example, in the DRC, in addition to the HIV law, reform efforts are also targeting the Penal Code, Law on Protection of Children, and Family Code, all of which were identified as problematic to HIV efforts in the LEA [24].

## Discussion and conclusion

The conduct of LEAs, NDs, action planning and capacity building can contribute to changes in knowledge and attitudes directly relevant to reforming laws that are found to be harmful. Multiple project countries stated that additional efforts were needed to enforce existing human rights and constitutional guarantees identified through the LEA and ND processes [5]. A nationally-owned action plan with in-built mechanisms for accountability, created from priority-setting during the ND, is a key element to promoting appropriate actions moving forward.

These are not the only activities that contribute towards legal change, but these lessons are foundational for creating movement towards such change. This project learning is not linear, nor will these issues play out in the same way everywhere. Exactly how rights will be implemented or how LEAs and capacity building activities carried out will depend on the local context, stakeholders, capacity and collaborations. It will, however, be useful for all such legal reform projects to give significant attention and resources to each of these areas in their efforts towards legal reform as part of the HIV response.

The UNDP project exemplifies global recognition of the importance of strengthening legal environments to improve national HIV responses. The work led to a multitude of positive practice and policy changes across different countries. Given the complexities of politics and law-making, such positive changes, while no guarantee of legal change to come, are significant for improving HIV responses along the way [1, 5].

It is critical to understand the incremental steps, both positive and negative, along the pathway to legal change to effectively address legal environments and their impact on HIV. It is also important to track negative changes, such as the addition of laws criminalizing HIV transmission, to understand the forces behind them and inform further advocacy efforts. A range of shorter-term goals can be identified as interim markers of positive change, and recognition of these can maintain momentum. These steps can serve as key project activities and provide interim indicators of progress towards the goal of improved structural factors and national HIV responses. Tracking the issues raised in the results section above and incorporating relevant indicators into project monitoring and evaluation frameworks can help understand progress towards legal change within project timeframes and inform project adjustments as required.

Systematic attention to human rights is key; it can help ground the approach taken to the steps along the way and ensure local ownership, nuanced understanding of the political landscape, attention to key populations, and accountability for action or inaction. Continuing to make progress in understanding the signs of progress along the causal pathway towards changes in the legal environment that can positively affect HIV-related outcomes will be important in any such work going forward.
